# Modified low-dose second window indocyanine green technique improves near-infrared fluorescence image-guided dermatofibrosarcoma protuberans resection: A randomized control trial

**DOI:** 10.3389/fsurg.2022.984857

**Published:** 2022-11-10

**Authors:** Lei Cui, Gao F. Wang, Xin Li, Yu Q. Song, Wen W. Pu, De K. Zhang, Wei Q. Jiang, Ya Q. Kou, Zhao Q. Tan, Ran Tao, Yan Han, Yu D. Han

**Affiliations:** ^1^ Department of Plastic and Reconstructive Surgery, Plastic Surgery Hospital (Institute), CAMS, PUMC, Beijing, China; ^2^ Department of Plastic and Reconstructive Surgery, 1st Medical Center of Chinese PLA General Hospital, Beijing, China; ^3^ Pathology Department, 1st Medical Center of Chinese PLA General Hospital, Beijing, China; ^4^ Transformation Laboratory, 1st Medical Center of Chinese PLA General Hospital, Beijing, China; ^5^ Radiology Department, 1st Medical Center of Chinese PLA General Hospital, Beijing, China

**Keywords:** intraoperative near-infrared fluorescence imaging, second window indocyanine green technique, dermatofibrosarcoma protuberans, tumor radical resection, tumor-to-background ratios

## Abstract

**Objective:**

Conventional second window indocyanine green (SWIG) technique has been widely attempted in near-infrared fluorescence (NIRF) imaging for intraoperative navigation of tumor radical resection. Nevertheless, the overuse of indocyanine green (ICG) led to an increased risk of drug lethal allergy and high medical cost. This prospective study was to explore clinical application of modified low-dose SWIG technique in guiding dermatofibrosarcoma protuberans (DFSPs) radical resection.

**Method:**

Patients with DFSPs were randomly assigned to control and experimental group. The ICG was injected intravenously 24 h before surgery, at a dose of 3.5 mg/kg in the control group and 25 mg/patient in the experiment group, respectively. Intraoperative NIRF imaging included serial views of gross tumor, tumor bed and cross-sectional specimen.

**Results:**

Although NIRF imaging of gross tumor and tumor bed in the experimental group demonstrated similar sensitivity and negative predictive value, the specificity and positive predictive value were obviously higher compared to control group. The tumor-to-background ratios of cross-sectional specimens in the experimental group was significantly higher than in the control group (*P* = 0.000). Data in both groups displayed that there was a positive correlation of tumor size in cross-sections between integrated histopathologic photomicrographs and NIRF imaging of specimen views (*P* = 0.000). NIRF imaging of cross-sectional specimens had a significant decrease in time cost, and an increase in the ability of examining more surgical margins (*P* = 0.000).

**Conclusion:**

This is the first study to demonstrate that a low-dose SWIG technique could improve the accuracy of near-infrared fluorescence image-guided dermatofibrosarcoma protuberans resection.

**Clinical Trial Registration:** ChiCTR2100050174; date of registration: August 18, 2021 followed by “retrospectively registered”

## Introduction

Dermatofibrosarcoma protuberans (DFSP) originating from the dermis or subcutis is a relatively rare yet stubborn tumor, characterized by infiltrative growth, high risk of local recurrence and extremely uncommon distant metastasis. To improve local control, it is preferably recommended that tumor *en bloc* resection should be performed to acquire adequate surgical margins of at least 2 cm according to conventional histopathology (1–3). Theoretically, three-dimensional micrographic surgery is more preponderant in terms of acquiring negative margins (4, 5). Given labor factor and examining time, however, it is impracticable to perform this procedure in clinical settings, especially in the case of huge tumors with a diameter greater than 2 cm.

With the development of intraoperative navigation, a variety of technologies, such as BrainLab intraoperative 3-dimensional magnetic resonance imaging (MRI) (6, 7), Raman spectroscopy (8), intraoperative computerized tomography (CT) (9), and Cerenkov luminescence imaging (CLI) (10), have been investigated to guide complete tumor removal, as well as locate small or metastatic lesions. In the past decades, the intraoperative MRI or CT surgical units are applied more and more widely in the resection of deep lesions, whereas these approaches were seldom used in the surgery of cutaneous malignant tumor because of sophisticated installation and operating procedure. Nowadays, most of Raman spectroscopy and CLI are at the preclinical stage. Moreover, CLI involves some controversial issues of radiation protection and occupation exposure, which limits the popularity of its clinical practice. In contrast, near-infrared fluorescence (NIRF) imaging using indocyanine green (ICG), a fluorescent contrast dye approved by Food and Administration (FDA), has been greatly broadened from ophthalmology (11), liver surgery (12–14), sentinel lymph nodes biopsy (15), and flap transfer (16) to intraoperative navigation in all sorts of tumor resections. In 2009, Kokudo et al. (17) happened to discover that hepatocellular carcinoma fluoresced strongly when these patients received a routine liver function test by intravenous injection of ICG prior to the surgery, which resulted in a clinical trial to investigate this novel fluorescent imaging techniques. They intravenously injected ICG at a dose of 0.5 mg/kg at least 24 h before surgery. Their study demonstrated that intraoperative real-time ICG-fluorescent imaging enables the highly sensitive identification of hepatocellular carcinoma and metastatic lesions in livers, improving the accuracy of liver resection and surgical staging. ICG is not tumor specific, non-cancerous tissue surrounding the tumor displays fluorescent signal as well. Hence, second window ICG (SWIG) technique based on the enhanced permeability retention (EPR) effect was put forward to improve tumor background ratios (TBRs) by altering the time and dose of ICG injection (18, 19). Nowadays, SWIG approach of high-dose (2.5 mg/kg-5 mg/kg) ICG intravenous infused 24 h prior to surgery has been widely attempted in intraoperative NIRF imaging for identifying tumor margins or discriminating occult cancerous lesions (19–26).

Nevertheless, the dose of ICG was overused on patients and violated package insert in previous clinical trials of SWIG approach, which led to an increased risk of drug lethal allergy and extremely high medical cost. In contrast to excessive dose of ICG, we designated 25 mg ICG for each patient as low-dose approach. Our current research was to explore clinical application of modified low-dose SWIG technique in guiding DFSP radical resection.

## Methods

Patients with DFSPs in heads, extremities or trunk were recruited between October 2019 and May 2022**.** The main exclusion included seafood/iodine allergy, hyperthyroidism, pregnancy, myasthenia gravis, or acute severe hypertension. The study, registered under chictr.org.cn (ChiCTR2100050174), was approved by the Medical Ethics Committee of the Chinese PLA General Hospital and rigidly obeyed the principles of the Declaration of Helsinki. All informed consent was obtained before ICG administration.

## NIRF image procedure

**Figure 1:**
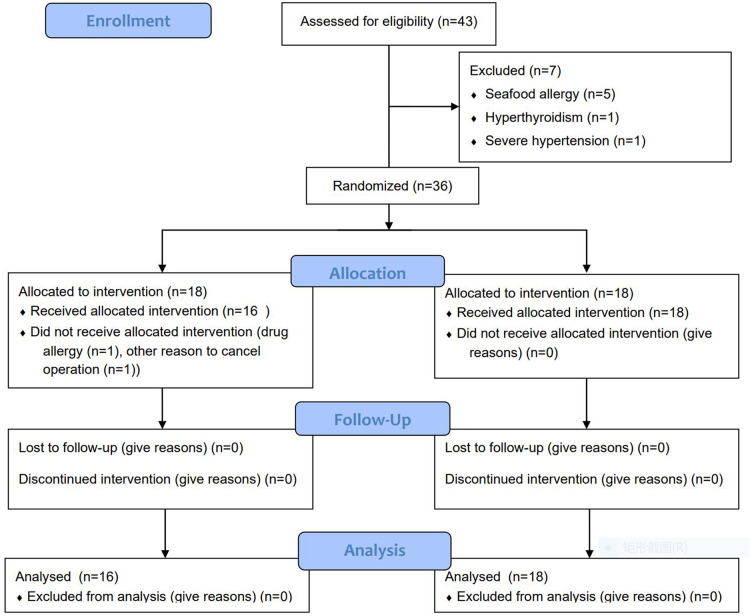
The diagram of enrollment and allocation for this randmomized controlled trial.

The patients enrolled in current research were randomly divided into control and experimental groups with a 1:1 allocation ratio based on block randomization by IBM SPSS software version 20.0 (IBM Corp., Armonk, NY) (Figure 1). The programs of ICG (2.5 mg/ml) (C43H47N2O6S2·Na; Danton Pharmaceutical Company) intravenous infusion were as follows: In the control group, the plan of ICG injection was 3.5 mg/kg 24 h before surgery, which referred to the dosage applied in previous research (2.5 mg/kg-5mg/kg). In the experimental group, the designed ICG injection was 25 mg/patient 24 h before surgery. The standard administration protocol included allergy test, antiallergic precondition and intravenous infusion of ICG. 8 mg ICG was injected *via* median cubital vein. After 20 min, if the test result was negative, the antiallergic preconditions consisting of 10 mg dexamethasone sodium phosphate and 20 mg diphenhydramine were administrated. Finally, the surplus ICG was infused intravenously. The adverse effects of ICG were recorded.

**Figure 2:**
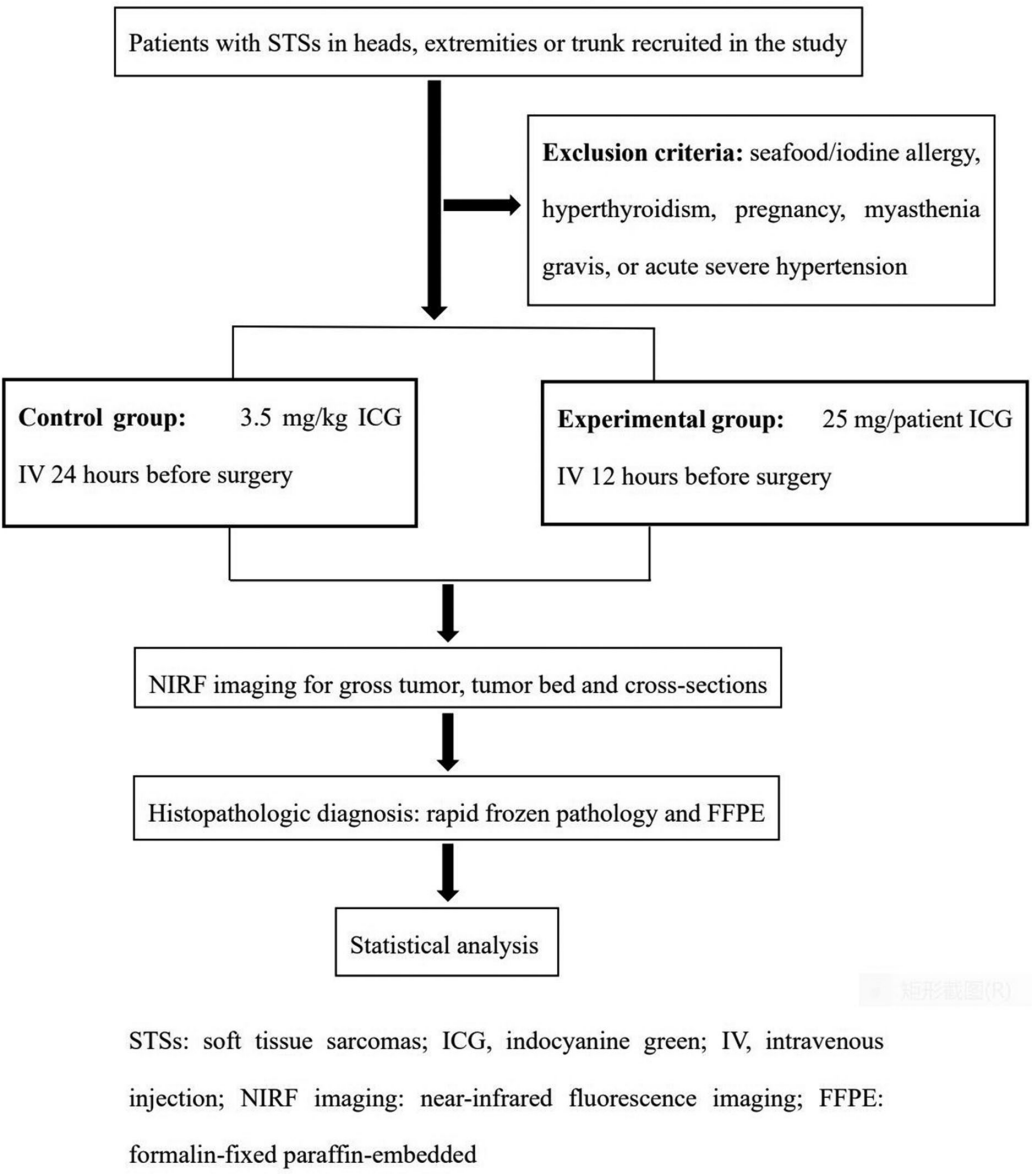
The workflow of study.

Fluorescent camera system (ARGOS NIR-300PT, Jinan Xianweizhineng Technology Co., Ltd) was applied to scan the tumor, with an 785-nm laser excitation source and 830 to 900-nm emission filter. The computer screen with a 3,820 × 2,160 pixel video resolution could offer white light view and pseudocolor view. The probe was positioned 20 cm above the operative area. Initially, the preoperative fluorescence imaging was referred to as “**gross tumor view**”. Patients underwent radical tumor resections at a safe margin distance of 2 cm from tumor fluorescent signals. After tumor removal, NIRF imaging was captured by visualizing surgical cavity again to detect residual cancerous tissues, which was named as “**tumor bed view**”. The suspicious lesions discerned by high fluorescent signals were further removed and diagnosed by histopathologic examinations. If there was no fluorescent signal in tumor beds, surgeons were designated to collect tissue as negative tumor base for final histopathologic examination. Sequentially, based on pathologic protocols, representative areas with close margins and the greatest horizontal and perpendicular dimensions of the gross specimens were cut transversely into cross-sections with the thickness of 10 mm in the vertical direction to the skin. Each cross-section was inspected using NIRF probe. The final ICG fluorescence imaging was defined as “**specimen view**”, which offered the information of TBRs and tumor size in cross-sectional specimens. The fluorescent imaging of “specimen view” could not affect the range of tumor resection in accordance to panel's recommendation. However, all cross-sections were evaluated by the final pathologic diagnoses. During this phase, the scanning time and number of surgical margins assessed by multiple cross-sections were also compared with conventional rapid frozen pathology. Figure 2 shows a workflow of the present study.

## Histopathologic diagnosis

After completing “specimen view”, the dissected specimen was retrieved to an intact one by suturing. In addition to routine specimen collection, each cross-section was subsequently cut into multiple small portions, the purpose of which was to measure tumor size by assembling into a complete photomicrograph by Adobe Photoshop (PS CC2020, Adobe Systems Incorporated, San Jose, California, USA). Pathological assessment included histologic type, tumor grade and size, and resection margin status. The surgical margins was reported based on the American Joint Committee on Cancer (AJCC) classification/R systems (27), which defines margins as negative (R0), microscopically positive (R1) and macroscopic tumor contamination (R2). Pathologic diagnoses were performed by two experts majoring in soft tissue sarcoma**.**

## Image analysis

ImageJ (https://imagej.nih.gov/ij/; National Institutes of Health, Bethesda, MD, USA) was used to analyze the TBRs and tumor size in fresh resected cross-sections from NIRF images of specimen views. Region of interest (ROI) size was defined as 100 mm^2^, given that the diameter of tumor in our study was more than 20 mm and the distance between tumor margin and surgical margin was more than 20 mm. The TBRs were obtained by manually drawing ROI areas of tumor and normal tissue to measure mean grey values on black-and-white images of cross-sectional specimens.

## Statistical analysis

PASS software was used to calculate sample size. Fisher's exact test, Mann-Whitney *U* test or independent *t*-test were used to compare baseline characteristics between two groups. Two-by-two contingency tables were used to calculate the sensitivity, specificity, positive predictive value (PPV) and negative predictive value (NPV) with 95% confidence intervals. TBRs of cross-sectional specimens between two groups were compared by Mann-Whitney *U* test. Spearman correlation analyses were performed to compare the maximum tumor dimension in cross-sections between integrated H&E slides and NIRF imaging of specimen views among respective group. An independent *t*-test was used to analyze the examining time between rapid frozen pathology and NIRF imaging of specimen view. Mann-Whitney *U* test was used to compare number of surgical margins between rapid frozen pathology and NIRF imaging of specimen view. All statistical analyses were performed using IBM SPSS software version 23.0 (IBM Corp., Armonk, NY).

## Results

### Patient characteristics

The needed sample size in this prospective two-arm randomized study was estimated by an alfa-value of 5%, a power of 85% and a difference in TBR of 1 between the two groups. The estimated sample size was 12 patients in each group. Hence, a total of 36 patients were enrolled in our study. 1 patient in the control group developed a skin rash during ICG administration. One operation was cancelled duo to nonmedical reason in the control group. At last, there were 16 patients in the control group and 18 patients in the experimental group (Figure 1). There were no significant differences between two groups as regards age (*P* = 0.351), gender (*P* = 1.000), histologic grade (*P* = 1.000), tumor location (*P* = 0.943), tumor size (*P* = 0.76) and body mass index (BMI) (*P* = 0.91). Comparison of patient demographics between two groups are summarized in Table 1.

**Table 1:** Comparison of patient demographics between 2 groups.

Variable	Control group (*n* = 16)	Experimental group (*n* = 18)	*P* Value
Age, years, median (interquartile range)	46.50 (41.00, 53.50)	41.25 (39.50, 54.30)	0.351
Gender, female: male ratio	7:9	8:10	1.000
Histologic grade (G), low: high ratio	8:8	8:10	1.000
Tumor location, *n*			0.943
Anterior trunk	3	3	
Posterior trunk	6	7	
Lower extremity	2	4	
Upper extremity	4	4	
Head	1	0	
Tumor size, largest diameter, mean ± SD, in centimeter	5.78 ± 1.53	5.83 ± 1.46	0.76
Body mass index (BMI), mean ± SD	24.14 ± 2.80	24.25 ± 2.81	0.91

### Analysis of NIRF imaging

16 DFSPs in 16 patients (100%) in the control group displayed significant NIRF imaging on gross tumor views. Four positive fluorescent signals in 4 of 16 patients were detected on tumor bed views, whereas these 4 lesions were confirmed as normal tissue or fibrous tissue based on histopathologic diagnoses. Based on the final pathology, the sensitivity, specificity, PPV, and NPV of NIRF imaging were 100%, 75.0%, 80.0%, 100%, respectively (Table 2). All tumors (100%) in the experimental group demonstrated positive NIRF imaging on gross tumor views. Three positive fluorescent signals from tumor bed views were observed in 3 of 18 patients. According to the final histopathology, two of these three suspicious positive tissue were diagnosed as clusters of spindle-shaped CD34-positive neoplastic cells. The other one was normal tissue. In the experimental group, the sensitivity, specificity, PPV, and NPV of NIRF imaging were 100%, 93.8%, 95.2%, and 100%, respectively (Table 2).

**Table 2:** Test characteristics of intraoperative NIRF imaging for gross tumor and tumor bed in the control group.

Group	NIRF imaging	Pathologic diagnosis		Test statistic (95% CI)			
		Positive	Negative	Sensitivity	Specificity	PPV	NPV
Control group	Positive	16	4	100% (75.9%–100%)	75.0% (47.4%–91.7%)	80.0% (55.7%–93.4%)	100% (69.9%–100%)
	Negative	0	12				
Experimental group	Positive	20	1	100% (80.0%–100%)	93.8% (67.7%–99.7%)	95.2% (74.1%–99.8%)	100% (74.7%–100%)
	Negative	0	15				

**Figure 3:**
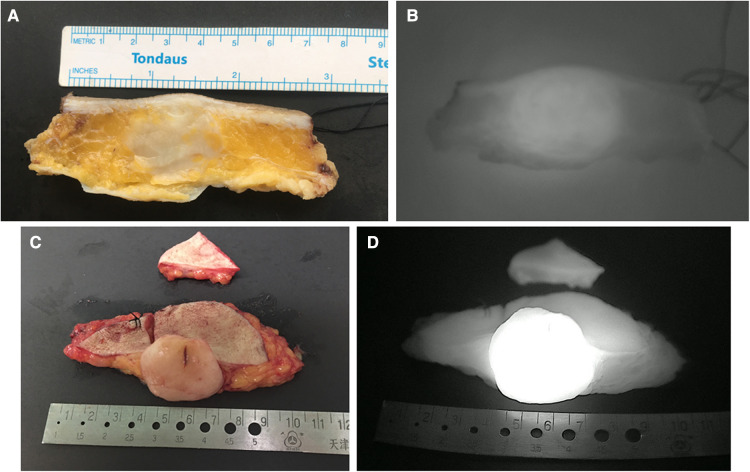
Representative images of cross-sectional specimen. (**A**) Ex vivo brightfield image and (**B**) ex vivo NIRF image of representative cross-section in the control group. (**C**) Ex vivo brightfield image and (**D**) ex vivo NIRF image of representative cross-section in the experimental group.

To identify the optimal SWIG technique, we compared the fluorescent intensity of cross-sections in “specimen views” between these two groups. The difference in TBRs of cross-sectional specimens between these two groups was statistically significant (3.90 (3.45–4.45) in the control group vs. 4.90 (4.60–5.50) in the experimental group; *P* < 0.001). It was noted that DFSPs in the experimental group showed significantly more intensive fluorescent signals within tumor sites. On the other side, tumor size calculated by the largest diameter did not differ between these two groups (5.78 ± 1.53 in the control group vs. 5.83 ± 1.46 in the experimental group, *P* = 0.76). The data from both groups displayed that there was a positive correlation of maximum tumor size in cross-sections between integrated H&E photomicrographs and NIRF imaging (rs = 0.989, *P* < 0.001, for the control group; rs = 0.996, *P* < 0.001, for the experimental group). Representative cases in each group are shown in Figure 3.

To compare with rapid frozen pathology, some corresponding parameters of intraoperative “specimen view” were recorded. Firstly, there was a statistically significant decrease in examining time between rapid frozen-section diagnoses and NIR fluorescence imaging of specimen views (47.85 ± 10.82 min vs. 10.21 ± 3.46 min, *P* < 0.001). In addition, the number of surgical margins evaluated by NIRF imaging of specimen view was considerably increased compared with that by rapid frozen-section diagnosis (5.00 (5.00–5.00) vs. 13.00 (10.00–16.00), *P* < 0.001).

## Discussion

Intraoperative NIRF imaging by SWIG technique based on EPR effect has been investigated in the navigation for tumor radical resection in recent years. The EPR effect hypothesizes that after a certain time period, high-intensity ICG is still retained in tumor cells due to enhanced permeability of tumor vasculature, whereas fluorescent imaging of normal tissue has disappeared. Singhal et al. (18) analyzed NIRF imaging of murine tumor models, that infused with various doses from 0.71 to 10 mg/kg of ICG at different timepoints between immediately after injection up to 72 h later. They concluded ICG for NIRF imaging of non-hepatic solid tumors was optimal when dosed at 5 mg/kg and 24 h prior to surgery. Subsequently, surgeons performed clinical researches to observe NIRF imaging of a variety of solid tumors using SWIG technique. In 2016, the first-in-human study (20) enrolled 15 patients infused with a 5 mg/kg dose of ICG 24 h preoperatively. Their results displayed that 12/15 gliomas were visualized with the NIRF imaging, with a sensitivity of 98% and specificity of 45% to confirm malignant areas in gadolinium-enhancing specimens. Similar results were later validated in brain metastases (24), pituitary adenomas (26), and intracranial meningiomas (21), pulmonary metastasectomy (23), breast lumpectomy (28) and other malignant tumors, in which the doses of ICG have varied between 5 mg/kg to 2.5 mg/kg. Newton et al. (22) stratified patients by tumor histology and investigated the optimal dose of ICG in second window technique. They demonstrated that higher dose ICG (4–5 mg/kg) is optimal for intraoperative NIRF imaging of lung cancers and lower dose ICG (2–3 mg/kg) is superior for non-primary lung cancers caused by decreased background fluorescence signal. Based on research evidence and empirical data, we assume that the optimal dose of ICG injection correlates with tumor characteristics and parameters of NIRF imaging device.

DFSP is a common type of soft tissue sarcomas (STSs). In theory, ICG as macromolecule is liable to be accumulated within the DFSP, characterized by capsule or pseudocapsule neoplasm surrounded by avascular subcutaneous fat tissue. At present, radical resection with at least 2 cm surgical margins is primary treatment to minimize the risk of local recurrence. To our knowledge, this is the first clinical trial to display intraoperative NIRF fluorescence imaging of DFSP. We designed traditional SWIG technique as control group. Firstly, we compared the low-dose SWIG technique with conventional high dose group in terms of the accuracy of identifying malignant tissue by NIRF views of gross tumor and tumor bed. NIRF imaging in the experimental group demonstrated similar sensitivity (100% vs. 100% in the control group), higher specificity (93.8% vs. 75.0% in the control group), increased PPV (95.2% vs. 80.0% in the control group) and similar NPV (100% vs. 100% in the control group). In both groups, all gross tumors were detected by NIRF imaging. 3 positive fluorescent signals were observed from tumor bed views in experimental group, two of which were confirmed as residual DFSP tissue. In contrast, 4 positive fluorescent signals in tumor beds were validated as non-malignant tissue in the control group. Therefore, we assumed that low-dose SWIG technique appeared to be more sensitive in visualizing tumor bed. Secondly, we analyzed fluorescent signals in cross-sectional specimens. The TBRs of dissected cross-sections in the experimental group was significantly higher than in the control group (4.90 (4.60–5.50) vs. 3.90 (3.45–4.45), *P* = 0.000). Hence, we conclude that the low-dose SWIG technique (25 mg/patient) displayed an improved performance of identifying DFSP lesions by intraoperative NIRF visualization system.

Our NIRF visualization system has more sensitive femtomolar probe detecting fluorescent dye at a concentration of 300 pm/L, the light source output of three homologous wavelength and the camera with three channels. The highly sensitive NIRF optical system decreases the dosage of contrast agent from 5 mg/kg to 0.2 mg/kg, which is one of the reasons why we chose 25 mg ICG in one ampoule as the dose in the experimental group. Besides, the images are processed to reduce background signal by a convolutional neural network approach. Therefore, compared to previous reports, our NIRF imaging system is capable of producing more favorable outcome than conventional SWIG technique.

Although the incidence of lethal complication is less than 0.1%, the iodine, a main component of ICG, is liable to induce a severe allergic reaction, even shock and death. Since the number of patients in our study was small, we didn't analyze the incidence of drug allergy. However, 1 patient had a mild skin rush during ICG administration in the control group. Take a patient with a weight of 65 kg as an example. The ICG in the control group costs 1,458 RMB (162 × 9), and that in the experimental group spends 162 RMB, which is obviously a very different cost. Given that drug safety, convenience of drug administration, and inpatient cost, 25 mg ICG as a single bolus has also a distinct advantage over high-dose infusion (2.5–5.0 mg/kg ICG).

**Figure 4:**
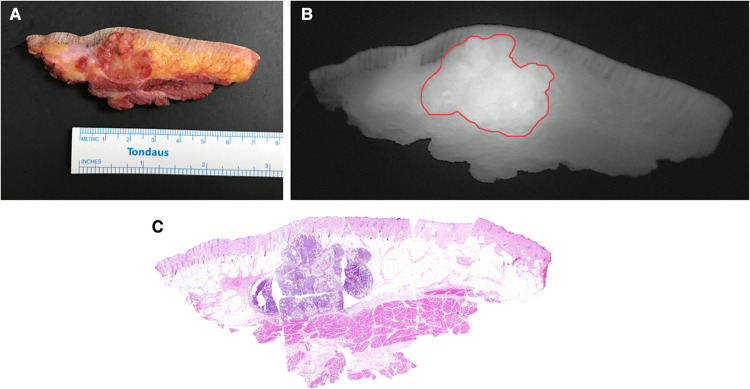
Cross-sectional specimen in a case of apocrine carcinoma. (**A**) Ex vivo brightfield image. (**B**) Ex vivo NIRF image. (**C**) Intact H&E photomicrograph integrated from multiple sections.

Even so, there are still some issues to address. Firstly, 25 mg ICG are suitable for patients with normal body mass index (BMI). The individualized dosage in modified SWIG technique should be verified in the following study. Secondly, the inclusion criteria stipulated that only patients with DFSPs were recruited in our study. In fact, intraoperative NIRF imaging using this low-dose SWIG technique might play a positive role in visualizing other malignant soft tissue tumors, such as other types of sarcomas and cutaneous squamous carcinomas. Figure 4 shows a case of apocrine carcinoma. Fluorescent signal demonstrated remarkable consistency with the contour of tumor in H&E slides. In the further, we will investigate this modified low-dose SWIG method for guiding the resections in other types of malignant neoplasms originated from soft tissues. As far as we know, some research referring to this low-dose SWIG technique in the field of other cancer therapy, such as lung cancer and breast cancer, is also ongoing. Especially for breast cancer locating in trunk and surrounded by adipose tissue, this modified approach is very meaningful in the surgical navigation for lumpectomy.

Besides a great deal of confidence in the prospect of low-dose SWIG technique for NIRF imaging, we also discovered that intraoperative NIRF imaging with SWIG technique could evaluate resected specimen in a more efficient, thorough, and quantitative manner.

At present, some problems in traditional histopathologic diagnosis remain to be solved. Firstly, there are several classification schemes of surgical margins in soft tissue sarcomas, such as the Musculoskeletal Tumor Society (MSTS) classification (29), the American Joint Committee on Cancer (AJCC) classification (27), and the Toronto Margin Context Classification (TMCC) (30), which involve the information of margin status, anatomic barrier and metric distance. Whichever classification is applied, the histopathological examinations just inspect few topical margins, which is obviously inadequate for assessing gross resection specimen, especially for those huge tumors. Moreover, intraoperative frozen-section diagnosis is incapable of offering precise margin distance and prolongs operative time to some degree.

In the present study, we dissected surgical specimen into multiple cross-sections and scanned cross-sectional specimens in sequence using NIRF imaging with SWIG technique before rapid frozen-section histopathology. There was a significant increase in the number of surgical margins assessed by NIRF imaging of specimen views compared to that by rapid frozen-section diagnoses (13.00 (10.00–16.00) vs. 5.00 (5.00–5.00), *P* = 0.000). The average examining time of rapid frozen-section diagnoses was 47 min, which was evidently longer than intraoperative NIRF imaging of specimen views with a mean time of 10 min. Technically, since the NIRF imaging has the advantage of rapid scanning, it is capable of inspecting the whole resected specimen within a few minutes. This efficient approach of evaluating gross surgical specimen could increase the number of positive margins along with a great amount of cross-section scanning. Additionally, NIRF imaging can offer more detailed information about tumor size and margin distance in every cross-section, which is impossible in rapid frozen diagnosis. In our study, the data in both groups displayed that there was a positive correlation of largest diameters of malignant tissue in cross-sections between integrated H&E photomicrographs and NIRF imaging of specimen views (rs = 0.989, *P* = 0.000, for the control group; rs = 0.996, *P* = 0.000, for the experimental group). Although margin distances were not analyzed in our research, the measurement of margin distance is feasible and beneficial for surgeons, especially when resected margins are suspicious.

**Figure 5:**
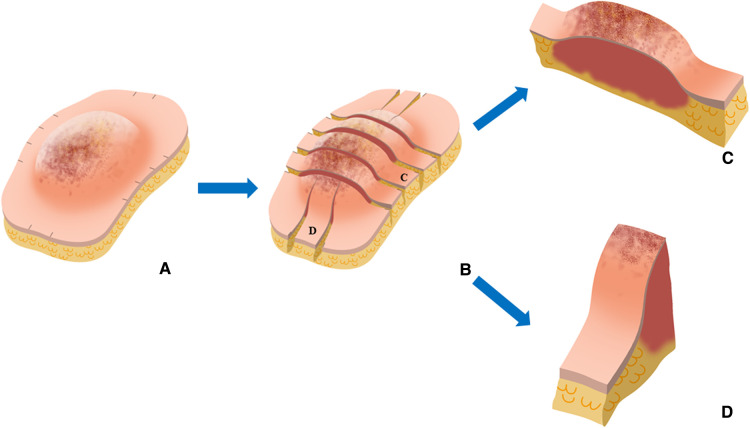
The schematic diagram of inspecting intact surgical specimen in the future.

We limited the quantity of dissected cross-sections at the current stage, so that the pathologists could assess the intact tumors before sampling. We plan to dissect surgical specimens from comprehensive and multidimensional viewpoints in the future study. Furthermore, we will proceed with investigating whether intraoperative NIRF imaging has potential in assisting pathologists with improving the performance of histopathologic diagnosis. Figure 5 shows a schematic diagram of inspecting intact surgical specimen in the future. For instance, under the direction of specimen views, how to efficiently assess gross surgical specimens, how to accurately harvest suspicious margins, and how to acquire more detailed parameters of metric distance.

## Conclusions

To our knowledge, this was the first time that low-dose SWIG technique was investigated in NIRF imaging. The low-dose SWIG technique demonstrated improved accuracy of detecting tumor fluorescent signal in the intraoperative navigation of DFSP radical resection compared with conventional SWIG technique. Taking into consideration the benefit of drug safety, convenience of drug administration, and inpatient cost, we assume that this modified approach has a good prospect for extensive clinical application. Additionally, our research was the first to explore the potential application of intraoperative NIRF imaging for evaluating resected specimens. Further investigations of NIRF imaging using low-dose SWIG technique will focus on the following issues: a refinement in ICG dosage, broadening clinical application in a variety of malignant tumors, as well as optimizing NIRF imaging of specimen view for assisting pathologic diagnosis.

## Data Availability

The original contributions presented in the study are included in the article/Supplementary Materials, further inquiries can be directed to the corresponding author/s.

## Synopsis

This is the first study to demonstrate that a low-dose SWIG technique could improve near-infrared fluorescence image-guided dermatofibrosarcoma protuberans resection. Otherwise, intraoperative NIRF imaging with SWIG technique could evaluate resected specimen in an efficient, thorough, and quantitative manner.

## References

[1] Ugurel S Kortmann R Mohr P Mentzel T Garbe C Breuninger H S1 guidelines for dermatofibrosarcoma protuberans (DFSP)—update 2018. J Ger Soc Dermatol. (2019) 17(6):663–8. 10.1111/ddg.13849 31115967

[2] Molina A Duprat Neto J Bertolli E da Cunha IW Fregnani JHTG Figueiredo PHM Relapse in dermatofibrosarcoma protuberans: a histological and molecular analysis. J Surg Oncol. (2018) 117(5):845–50. 10.1002/jso.25039 29509956

[3] Sambri A Caldari E Fiore M Zucchini R Giannini C Pirini MG Margin assessment in soft tissue sarcomas: review of the literature. Cancers. (2021) 13(7):1687. 10.3390/cancers13071687 33918457 PMC8038240

[4] Wang S Ezaldein H Delost G Tripathi R Stamey C Neudecker M Safety and efficacy of mohs micrographic surgery in children and adolescents: a systematic review. Dermatol Surg. (2020) 46(7):880–4. 10.1097/dss.0000000000002282 31834072

[5] Charalambides M Yannoulias B Malik N Mann JK Celebi P Veitch D A review of mohs micrographic surgery for skin cancer. Part 1: melanoma and rare skin cancers. Clin Exp Dermatol. (2022) 47(5):833–49. 10.1111/ced.15081 34939669

[6] Di Carlo H Maruf M Massanyi E Shah B Tekes A Gearhart JP. 3-Dimensional magnetic resonance imaging guided pelvic floor dissection for bladder exstrophy: a single arm trial. J Urol. (2019) 202(2):406–12. 10.1097/ju.0000000000000210 30840542

[7] Scherer M Zerweck P Becker D Kihm L Jesser J Beynon C The value of intraoperative MRI for resection of functional pituitary adenomas-a critical assessment of a consecutive single-center series of 114 cases. Neurosurg Rev. (2022) 45(4):2895–907. 10.1007/s10143-022-01810-7 35567728 PMC9349072

[8] Zúñiga W Jones V Anderson S Echevarria A Miller NL Stashko C Raman spectroscopy for rapid evaluation of surgical margins during breast cancer lumpectomy. Sci Rep. (2019) 9(1):14639. 10.1038/s41598-019-51112-0 31601985 PMC6787043

[9] Bosma SE Cleven AHG Dijkstra PDS. Can navigation improve the ability to achieve tumor-free margins in pelvic and sacral primary bone sarcoma resections? A historically controlled study. Clin Orthop Relat Res. (2019) 477(7):1548–59. 10.1097/CORR.0000000000000766 31107331 PMC6999970

[10] Fragoso Costa P Fendler W Herrmann K Sandach P Grafe H Grootendorst M Radiation protection and occupational exposure on [Ga]Ga-PSMA-11 based cerenkov luminescence imaging procedures in robot assisted prostatectomy. J Nucl Med. (2021) 63(9):1349–56. 10.2967/jnumed.121.263175 34916249 PMC9454458

[11] Bousquet E Provost J Zola M Spaide R Mehanna C Behar-Cohen F. Mid-phase hyperfluorescent plaques seen on indocyanine green angiography in patients with central serous chorioretinopathy. J Clin Med. (2021) 10(19):4525. 10.3390/jcm10194525 34640543 PMC8509799

[12] Chen H Wang Y Xie Z Zhang L Ge Y Yu J Application effect of ICG fluorescence real-time imaging technology in laparoscopic hepatectomy. Front Oncol. (2022) 12:819960. 10.3389/fonc.2022.819960 35463377 PMC9020263

[13] Lin H Wang Y Zhou J Yang Y Xu X Ma D Tomoelastography based on multifrequency MR elastography predicts liver function reserve in patients with hepatocellular carcinoma: a prospective study. Insights Imaging. (2022) 13(1):95. 10.1186/s13244-022-01232-5 35657534 PMC9166923

[14] He K Hong X Chi C Cai C An Y Li P Efficacy of near-infrared fluorescence-guided hepatectomy for the detection of colorectal liver metastases: a randomized controlled trial. J Am Coll Surg. (2022) 234(2):130–7. 10.1097/xcs.0000000000000029 35213433

[15] Dumitru D Ghanakumar S Provenzano E Benson JR. A prospective study evaluating the accuracy of indocyanine green (ICG) fluorescence compared with radioisotope for sentinel lymph node (SLN) detection in early breast cancer. Ann Surg Oncol. (2022) 29(5):3014–20. 10.1245/s10434-021-11255-9 35000084

[16] Geierlehner A Horch R Ludolph I Arkudas A. Intraoperative blood flow analysis of DIEP vs. ms-TRAM flap breast reconstruction combining transit-time flowmetry and microvascular indocyanine green angiography. J Pers Med. (2022) 12(3):482. 10.3390/jpm12030482 35330481 PMC8950170

[17] Ishizawa T Fukushima N Shibahara J Masuda K Tamura S Aoki T Real-time identification of liver cancers by using indocyanine green fluorescent imaging. Cancer. (2009) 115(11):2491–504. 10.1002/cncr.24291 19326450

[18] Jiang JX Keating JJ Jesus EM Judy RP Madajewski B Venegas O Optimization of the enhanced permeability and retention effect for near-infrared imaging of solid tumors with indocyanine green. Am J Nucl Med Mol Imaging. (2015) 5(4):390–400. 26269776 PMC4529592

[19] Wu J. The enhanced permeability and retention (EPR) effect: the significance of the concept and methods to enhance its application. J Pers Med. (2021) 11(8):771. 10.3390/jpm11080771 34442415 PMC8402171

[20] Lee J Thawani J Pierce J Zeh R Martinez-Lage M Chanin M Intraoperative near-infrared optical imaging can localize gadolinium-enhancing gliomas during surgery. Neurosurgery. (2016) 79(6):856–71. 10.1227/neu.0000000000001450 27741220 PMC5123788

[21] Lee J Pierce J Thawani J Zeh R Nie S Martinez-Lage M Near-infrared fluorescent image-guided surgery for intracranial meningioma. J Neurosurg. (2018) 128(2):380–90. 10.3171/2016.10.Jns161636 28387632 PMC10985532

[22] Newton A Predina J Corbett C Frenzel-Sulyok LG Xia L Petersson EJ Optimization of second window indocyanine green for intraoperative near-infrared imaging of thoracic malignancy. J Am Coll Surg. (2019) 228(2):188–97. 10.1016/j.jamcollsurg.2018.11.003 30471345 PMC6348120

[23] Predina J Newton A Corbett C Shin M Sulfyok LF Okusanya OT Near-infrared intraoperative imaging for minimally invasive pulmonary metastasectomy for sarcomas. J Thorac Cardiovasc Surg. (2019) 157(5):2061–9. 10.1016/j.jtcvs.2018.10.169 31288365 PMC6625353

[24] Teng C Cho S Singh Y De Ravin E Somers K Buch L Second window ICG predicts gross-total resection and progression-free survival during brain metastasis surgery. J Neurosurg. (2021) 135(4):1–10. 10.3171/2020.8.Jns201810 33652417 PMC10998541

[25] Teng C Huang V Arguelles G Zhou C Cho SS Harmsen S Applications of indocyanine green in brain tumor surgery: review of clinical evidence and emerging technologies. Neurosurg Focus. (2021) 50(1):E4. 10.3171/2020.10.Focus20782 33386005

[26] Vergeer R Theunissen R van Elk T Schmidt I Postma MR Tamasi K Fluorescence-guided detection of pituitary neuroendocrine tumor (PitNET) tissue during endoscopic transsphenoidal surgery available agents, their potential, and technical aspects. Rev Endocr Metab Disord. (2022) 23(3):647–57. 10.1007/s11154-022-09718-9 35344185 PMC9156450

[27] Hermanek P Wittekind C. The pathologist and the residual tumor (R) classification. Pathol Res Pract. (1994) 190(2):115–23. 10.1016/s0344-0338(11)80700-4 8058567

[28] Keating J Tchou J Okusanya O Fisher C Batiste R Jiang J Identification of breast cancer margins using intraoperative near-infrared imaging. J Surg Oncol. (2016) 113(5):508–14. 10.1002/jso.24167 26843131 PMC11156255

[29] Enneking W Spanier S Goodman MA. A system for the surgical staging of musculoskeletal sarcoma. Clin Orthop Relat Res. (1980) 153:106–20. 10.1097/00003086-198011000-00013 7449206

[30] Gerrand C Wunder J Kandel R O’Sullivan B Catton CN Bell RS Classification of positive margins after resection of soft-tissue sarcoma of the limb predicts the risk of local recurrence. J Bone Joint Surg Br. (2001) 83(8):1149–55. 10.1302/0301-620x.83b8.12028 11764430

